# Neonatal stroke surveillance study protocol in the United Kingdom and Republic of Ireland

**DOI:** 10.1515/med-2022-0554

**Published:** 2022-09-05

**Authors:** T’ng Chang Kwok, Robert A. Dineen, William Whitehouse, Richard M. Lynn, Niamh McSweeney, Don Sharkey

**Affiliations:** Centre for Perinatal Research, Population and Lifespan Sciences, School of Medicine, University of Nottingham, Queen’s Medical Centre, Nottingham, NG7 2UH, United Kingdom; Radiological Sciences, Mental Health & Clinical Neuroscience, School of Medicine, University of Nottingham, Queen’s Medical Centre, Nottingham, NG7 2UH, United Kingdom; NIHR Nottingham Biomedical Research Centre, Nottingham, United Kingdom; Population, Policy and Practice Research and Teaching Department, University College London Great Ormond Street Institute of Child Health, London, WC1N 1EH, England; Department of Paediatrics and Child Health, Cork University Hospital, Wilton, Cork, T12 DC4A, Ireland; Centre for Perinatal Research, Population and Lifespan Sciences, School of Medicine, University of Nottingham, E floor, East Block, Queen’s Medical Centre, Nottingham, NG7 2UH, United Kingdom

**Keywords:** infant, neonatal stroke, population surveillance, perinatal stroke

## Abstract

Neonatal stroke is a devastating condition that causes brain injury in babies and often leads to lifelong neurological impairment. Recent prospective population studies of neonatal stroke are lacking. Neonatal strokes are different from those in older children and adults. A better understanding of its aetiology, current management, and outcomes could reduce the burden of this rare condition. The study aims to explore the incidence and 2 year outcomes of neonatal stroke across an entire population in the UK and Republic of Ireland. This is an active national surveillance study using a purpose-built integrated case notification-data collection online platform. Over a 13 month period, with a potential 6 month extension, clinicians will notify neonatal stroke cases presenting in the first 90 days of life electronically via the online platform monthly. Clinicians will complete a primary questionnaire via the platform detailing clinical information, including neuroimaging, for analysis and classification. An outcome questionnaire will be sent at 2 years of age via the platform. Appropriate ethics and regulatory approvals have been received. The neonatal stroke study represents the first multinational population surveillance study delivered via a purpose-built integrated case notification-data collection online platform and data safe haven, overcoming the challenges of setting up the study.

## 1 Introduction

Neonatal stroke is a devastating condition that causes brain injury and often leads to lifelong neurological impairment [1] including cerebral palsy [2,3] and global developmental delay in particular language and cognitive delay [4,5]. A common presenting symptom is focal seizures in the neonatal period with a later incidence of 15–25% of epilepsy depending on the extent and location of the injury [6]. Infants with neonatal stroke will continue to depend on healthcare services throughout childhood. It is estimated that each case of neonatal stroke will cost the healthcare services at least £26,000 (∼31,000 EUR; 34,000 USD) for the initial admission and a further £40,000 (∼48,000 EUR; 52,000 USD) during the first 5 years of life [7]. There will also be an increasing need for education services to support survivors of neonatal stroke [8]. The impact on family life and loss of economic productivity due to neonatal stroke is also known to be significant [9,10].

Neonatal strokes are different from strokes seen in older children and adults [11]. In babies, the brain is immature with greater capacity for plasticity in response to brain injury and hence greater potential for recovery [12]. However, the susceptibility of the developing newborn brain may make it more prone to injury leading to long-term impairment compared to older children. A better understanding of neonatal stroke will guide further research in identifying new interventions to prevent and treat neonatal stroke [13].

Although stroke is more common in the first 28 days of an infant’s life than in older children [14], the true contemporary incidence of neonatal stroke is still unclear. Previous Canadian [14] and UK studies [15] from over 20 years ago reported the incidence of neonatal stroke between 5.2–10.2 per 100,000 live births whereas a retrospective case-control study using neuroimaging [16] found an incidence of 37 per 100,000 live births. Previous retrospective case-control studies [17,18] were based on infants born in a province or small geographical area, with data collected over 20 years ago, limiting the generalisability of the findings.

As most clinicians may see only a few cases of neonatal stroke in their career, there is a variation in practice and guidance in the management of neonatal stroke [19]. This is unlike stroke in adults where much more is known. Hence, there is a need for an active surveillance study with follow-up to have a better understanding of the condition. The challenges of setting up a multinational electronic surveillance system as well as the regulatory approval process across multiple nations will also be discussed.

## 2 Aims

This active surveillance study aims to explore the burden of neonatal stroke in the UK and the Republic of Ireland (ROI) to better allocate resources and improve the care of babies with neonatal stroke.

### 2.1 Primary objectives

1. To estimate the incidence of stroke presenting in infants in the first 90 days of life in the UK and the ROI.
2. To determine the 2 year outcome of neonatal stroke, including neurodevelopmental outcome.

### 2.2 Secondary objectives

1. To analyse the proportion of the different types of neonatal stroke (arterial ischaemia, venous thrombosis, and haemorrhage) and their impact on presentation and outcome
2. To analyse known predisposing maternal and infant factors for neonatal stroke
3. To analyse the presentation of infants with neonatal stroke
4. To analyse the variation in clinical investigations used in neonatal stroke
5. To describe findings of investigations, particularly neuroimaging
6. To analyse the variation in management of neonatal stroke including the follow-up pathway.

## 3 Methods

### 3.1 Study design

**Figure 1:**
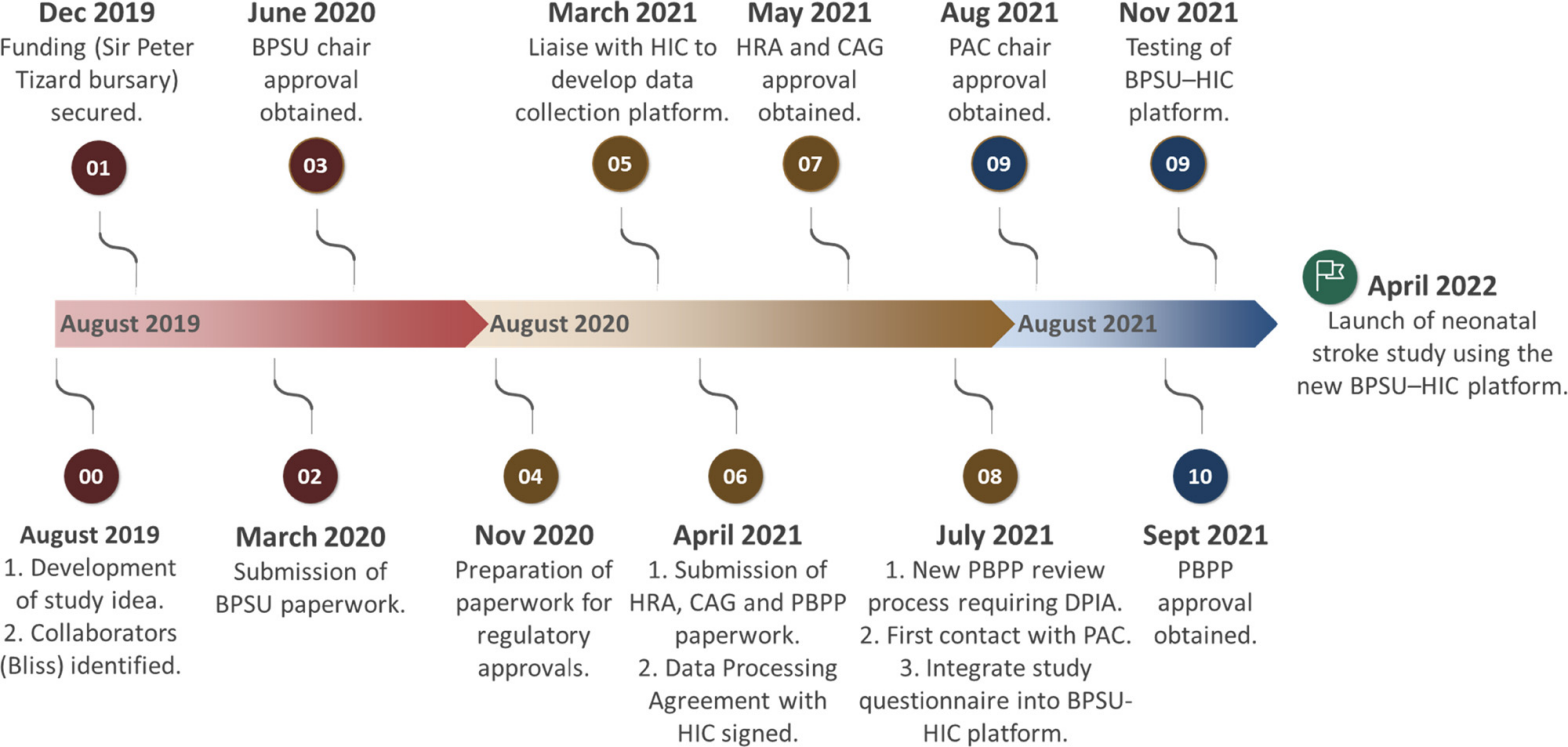
Timeline depicting the launch of the neonatal stroke surveillance study from the conceptualisation of the research idea. Study progress was halted for a year in March 2020 due to the COVID-19 pandemic. BPSU = British Paediatric Surveillance Unit. HIC = Health Informatics Centre. HRA = Health Research Authority. CAG = Confidentiality Advisory Group. PBPP = Public Benefit and Privacy Panel. DPIA = Data Protection Impact Assessment. PAC = Privacy Advisory Committee of Northern Ireland.

The study represents the first multinational population surveillance study using the British Paediatric Surveillance Unit (BPSU) active surveillance approach delivered via a purpose-built integrated case notification-data collection online platform and data safe haven, developed by BPSU in collaboration with the University of Dundee Health Informatics Centre (HIC) (Figure 1).

### 3.2 Active surveillance

Over a 13 month period, anonymised notifications of the stroke cases seen presenting in babies in the first 90 days of life will be provided to the BPSU using the BPSU orange e-Card delivered via the BPSU–HIC platform. A further 6 month extension to the 13 month period may be sought to allow the development and testing of the new reporting mechanism on the BPSU–HIC platform. The BPSU orange e-Card has a list of rare disorders under active surveillance which is sent electronically every month to over 4,200 British and Irish consultant paediatricians and other specialities. Information leaflets and study newsfeeds will be circulated to all clinicians receiving the BPSU orange e-Card during the study period.

Clinicians will indicate any cases of stroke seen by them in babies up to 90 days of age that meet the case definition, or “nothing to report” on the monthly BPSU orange e-card system and a response is expected. The “active surveillance” is an important feature of the surveillance scheme as it allows the compliance of the system to be continually monitored, ensuring good coverage of the paediatric surveillance scheme across the UK and ROI [20]. The reporting compliance rate in 2019 was 90% ensuring that the proposed study methodology will have high case ascertainment, validity, and reliability.

**Figure 2:**
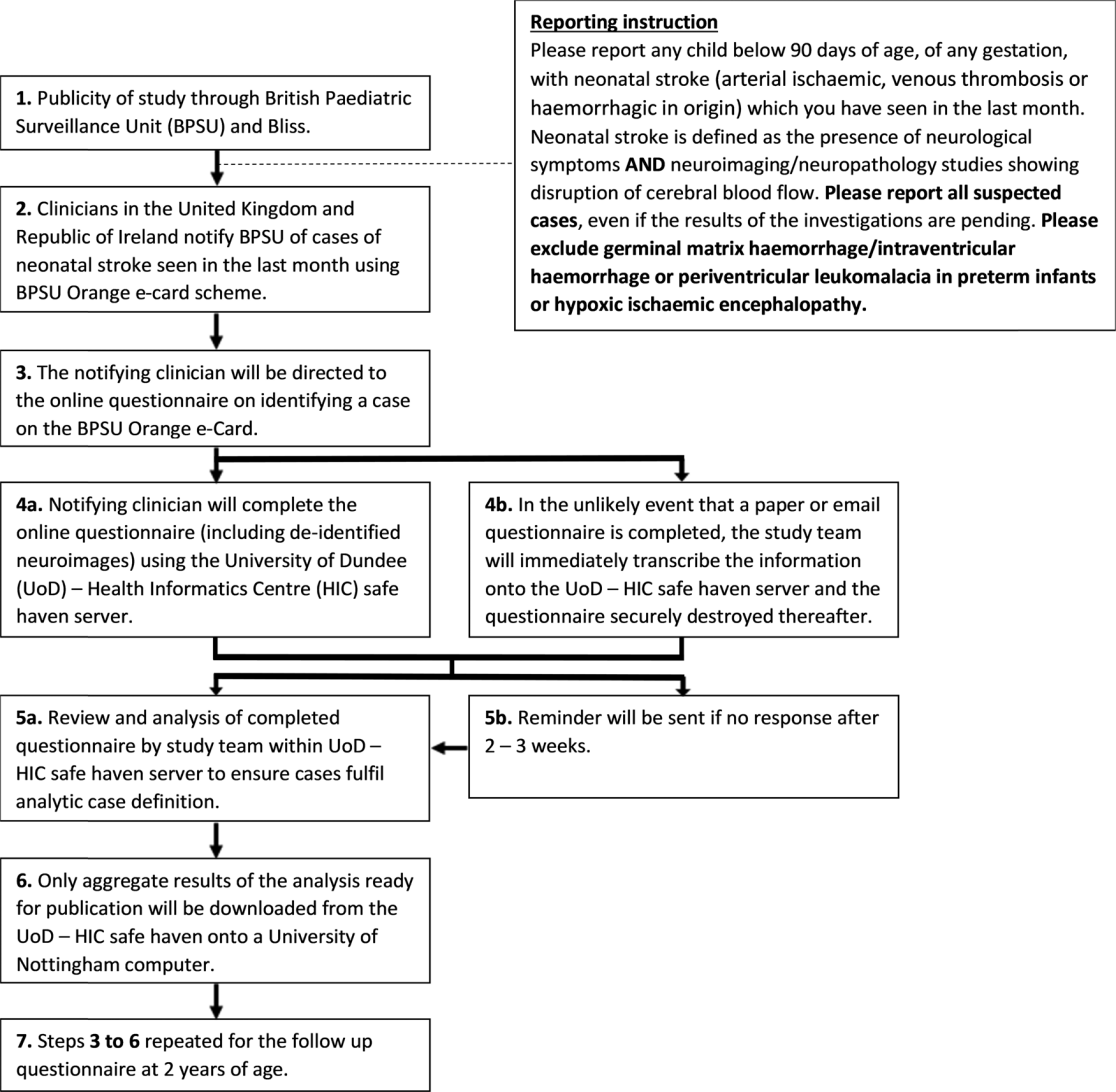
Flow diagram depicting the notification and data flow during the neonatal stroke surveillance study.

The notifying clinician will then be directed to an online initial questionnaire (Supplemental material 1) via the BPSU–HIC platform and complete the electronic questionnaire. The initial questionnaire (Supplemental material 1) will seek information about the presentation of neonatal stroke, demographic details, investigations including neuroimaging, and treatment received. On completion of the initial questionnaire, the reporting clinician will be asked to retain the patient details for a follow-up outcome questionnaire, which will be sent at 2 years of age (Supplemental material 2) through the BPSU–HIC platform (Figure 2). Each questionnaire will be date- and time-stamped on completion and specific to the notifying clinician. Separate questionnaires will be generated for Northern Ireland clinicians (Supplemental materials 3 and 4) due to differences in regulatory requirements.

### 3.3 Case definition

#### 3.3.1 Inclusion criteria

Any infant from birth till 90 days of age, of any gestation, with neonatal stroke is defined as:-

1. Developing any neurological symptoms (including seizure, neurological deficit, lethargy, abnormal tone, and poor feeding)
**AND fulfilment of either 2a OR 2b:**
2. a. Neuroimaging (such as MRI or CT) showing disruption or evidence of disruption of cerebral blood flow. The radiology findings include either:
  - Arterial ischaemia: defined as the presence of: (i) partial or complete occlusion of the cerebral artery(ies) in relation to a focal brain lesion; or (ii) brain lesion pattern with imaging that can only be explained by occlusion of a specific cerebral artery(ies);
**OR**
  - Venous thrombosis: defined as the presence of a thrombus within the cerebral vein(s) or venous sinus(es), with partial or complete occlusion.
**OR**
  - Haemorrhage: defined as the presence of a haemorrhage in the parenchyma of the brain.

b. Neuropathologic studies (such as post-mortem) showing disruption or evidence of disruption of cerebral blood flow

#### 3.3.2 Exclusion criteria

1. Hypoxic ischaemic encephalopathy
2. Germinal matrix haemorrhage/intraventricular haemorrhage or periventricular leukomalacia in preterm infants (defined as below 37 weeks of gestational age)
3. Metabolic injury such as kernicterus and hypoglycaemia
4. Encephalitis (bacterial and viral)
5. Accidental or non-accidental injury
6. Vascular anomalies such as arteriovenous malformation
7. Brain tumour

The eligibility criteria above are based on the 2006 international National Institute of Child Health and Human Development and the National Institute of Neurological Disorders and Stroke workshop [21] which defined perinatal stroke as “a group of heterogeneous conditions in which there is focal disruption of cerebral blood flow secondary to arterial or cerebral venous thrombosis or embolisation, between 20 weeks of foetal life through the 28th postnatal day, confirmed by neuroimaging or neuropathologic studies.” The radiological definition is based on Sebire et al. [22] used in the International Paediatric Stroke Study [2] and Govaert et al. [23]. The exclusion criteria will ensure that the study finding is comparable to other reports published nationally [24] and internationally [23].

As this study is focused on neonatal stroke rather than foetal stroke, the age definition for the study is from birth. The age range extends till 90 days of age to capture infants that present late with either neuroimaging or neuropathologic studies, but which suggested stroke occurring in the perinatal period. The number of cases between 28 and 90 days of age is expected to be minimal [14]. As we cannot be completely certain that the neuroimaging or neuropathologic studies accurately date the neonatal stroke to have occurred in the first 28 days, we will separate the cases of neonatal stroke presenting within 28 days and those presenting between 28 and 90 days.

### 3.4 Data collection

The online questionnaire was developed and delivered using the BPSU–HIC platform. Data entered by notifying clinicians will be stored within the HIC safe haven server which is ISO 270001 standard certified (https://www.dundee.ac.uk/hic/hicsafehaven/). If clinicians are unable to use the online system, questionnaires will be sent out in electronic format to the reporting clinicians via a secure email account (ISO 9001 Standards certified). As a last resort, a paper questionnaire form will be sent. It will be marked confidential and double enveloped for data collection with a self-addressed envelope to be returned to the research team via registered mail. In the unlikely event of email or paper questionnaires being completed, the study team will immediately transcribe the information onto the HIC safe haven server on behalf of the notifying clinicians and the questionnaires destroyed thereafter.

All the requested information will be obtained using data recorded in case notes and laboratory/radiology results only, without further direct contact or active participation with patients. No extra investigations will be sought. De-identified images from neuroimaging studies will be uploaded by the notifying clinicians onto the HIC safe haven server where possible. Images can also be sent using an encrypted CD, which are then uploaded on the HIC safe haven server immediately and the CDs destroyed by the research team. If the images are not available, a copy of the de-identified neuroimaging report will be requested.

Limited patient identifiable data will be obtained for study validity reasons to identify duplicate cases and track follow-up. Each case will be pseudonymised through a unique computer-generated identifier number. The minimal identifiable data collected for study validity purposes will be removed as soon as it is practically possible and will be kept separate from the rest of the anonymised clinical data linked by the unique identifier number. All patient identifiable data collected for identifying duplicate cases will be destroyed once the data collection process is complete.

All data will be collected using opt-out consent. Public information leaflets and flyers will be disseminated to participating clinicians and on the BPSU website. These will provide information about the neonatal stroke study as well as how families can opt-out of babies’ medical information from being used for research by informing their attending clinician. In the unlikely event that families decide to opt-out, a small amount of identifiable information will be held for the duration of the study to ensure that no further clinical information is collected.

### 3.5 Data management and security

All study data will be collected, analysed, and held securely on the HIC safe haven server, with access restricted by user login identifiers and passwords, which are encrypted using a one-way encryption method. The HIC safe haven server is ISO 270001 certified and is externally audited twice yearly to ensure compliance and is overseen by the HIC Information Governance Committee, with National Health Service (NHS) and legal membership. Only aggregate results of the analysis ready for publication will be downloaded from the HIC safe haven server. In keeping with standard practice [25], all anonymised data will be archived within HIC safe haven server for 20 years.

### 3.6 Analysis

Primary analyses will be based on all cases that meet the analytic case definition. The patterns of availability of data and timing of data collection will be summarised, including differentiation of fully, or partially completed variables from those completely missing. The aggregation of data variables will be described. Descriptive statistics will be presented as numbers and percentages with 95% confidence intervals estimated using the binomial exact method, for binary and categorical variables; mean values with standard deviations for normally distributed continuous variables; and medians with interquartile range for skewed continuous variables. Neuroimages collected will be reviewed by paediatric neuroradiologists within the research team (RAD) to identify concordance with the reported findings entered by reporting clinicians.

Estimation of incidence will include the first month of surveillance based on the date of diagnosis. Incidence rates will be estimated for the whole sample and by gender. Birth statistics used for the denominators for incidence estimation will be obtained by gender from:

- Office for National Statistics (England and Wales).
- National Records of Scotland (Scotland).
- Northern Ireland Statistics and Research Agency (Northern Ireland).
- Central Statistics Office (ROI)

**Patient and public involvement:** The neonatal stroke surveillance study was designed in partnership with Bliss, the national charity for families of babies born sick or premature as well as lay representatives of the BPSU scientific committee. Bliss is supportive of the research question and the need to collect information about neonatal stroke to improve the support and care provided to the babies and families affected.
**Regulatory approval:** The study had received ethics approval from the Nottingham 1 Research Ethics Committee (Reference 21/EM/0110) supported by the Health Research Authority on advice from the Confidentiality Advisory Group (England and Wales) (Reference 21/CAG/0061); the Public Benefit and Privacy Panel (Scotland) (Reference 2122-0006 Kwok); and the chair of the Privacy Advisory Committee (Northern Ireland).
**Dissemination:** The study is in the process of being registered with ClinicalTrials.gov. Study findings will be disseminated to the scientific community via peer-reviewed publications and conference presentations. Clinicians completing the 2 year questionnaire will be included as co-authors in future publications as part of the UK/Irish Neonatal Stroke study collaborative. The research team will also disseminate the findings to clinicians, parents/carers, and the public via the partnership with Bliss, British Association of Perinatal Medicine and British Paediatric Neurology Association. Anonymised aggregate data will be made available at the time of peer-reviewed scientific publications either via online supplementary material or the University of Nottingham research data repository.

## 4 Discussion

### 4.1 Sample size

It is estimated that our study will detect 150 cases of neonatal stroke per year. The Canadian paediatric stroke registry [14] reported the incidence of neonatal arterial ischaemic stroke to be 10.2 per 100,000 live births between January 1992 and December 2001, while the 2001 UK surveillance study [15] identified 31 neonatal stroke cases annually (5.2 per 100,000 live births [26]). Both studies [14,15] collected data over 20 years ago, when diagnostic imaging and reporting of neonatal stroke was not as advanced. The recent retrospective population-based cohort study using the national neonatal electronic database [24,27] reported a neonatal stroke incidence of 11–15 per 100,000 live births in England between 2012 and 2017. However, the study [24,27] excluded babies cared for outside the neonatal unit and only contains basic information on babies, without distinguishing between the different types of neonatal stroke and 2 year outcome. A population-based birth prevalence of disease-specific perinatal stroke in Alberta by Dunbar et al. [28] included prospective data collection between 2008 and 2017 and retrospective data between 1990 and 2008. They reported an overall prevalence of neonatal stroke of 1 in 1,100 of which 1 in 3,000 was neonatal arterial ischemic stroke (NAIS), 1 in 7,900 for presumed NAIS, 1 in 6,000 for periventricular venous infarction, 1 in 9,100 for cerebral sinovenous thrombosis, and 1 in 6,800 for neonatal haemorrhagic stroke [28].

### 4.2 Opt-out consent

The collection of a minimum dataset of identifiable patient information using opt-out consent is part of the conventional active surveillance methodology used by BPSU to prevent case ascertainment bias in the surveillance of a rare condition. Minimal identifiers are required to match duplicate cases reported to the study. If clinicians do not use the same reference number system, then more than one identifier, such as date of birth and gender, might be needed for matching. Some identifiers are also important pieces of clinical data in newborn infants, for example, the exact date of birth is required to calculate the age at diagnosis in days or weeks.

The incidence of neonatal stroke can only be estimated if information about all cases which occur within a specified period can be collected. As neonatal stroke is a rare condition, failure to obtain consent from only a few patients would make the incidence estimates inaccurate and under-ascertain cases. This may lead to incorrect conclusions being drawn, especially if the failure to obtain consent and data occurred in a single region or in babies with a less severe form of neonatal stroke. Furthermore, opt-out consent was found to be an acceptable method by clinicians and parents/carers in neonatal studies, including clinical trials [29].

### 4.3 Collaborative standardised regulatory approval

Section 251 of the NHS Act 2006 in the UK was established to allow confidential patient information to be used for essential NHS activities and important medical research where the use of anonymised information is not possible and seeking consent was not practical [30]. Hence, careful regulation of this process is needed to protect the interest of the patients and the public, while facilitating appropriate use of the information. At present, England and Wales, Scotland, as well as Northern Ireland, have different pathways to seek regulatory approvals for the use of patient information. A collaborative and standardised approach is needed across the four nations in the UK to avoid research wastage in completing different regulatory paperwork used in each nation, while ensuring the research is carried out to the highest standard in minimising the disclosure risk, while protecting confidentiality and interest of the patients and public.

### 4.4 Assessment of implementation of the new data collection platform

A Plan, Do, Check, and Act (PDCA) methodology will be used to assess the success and improve the new data collection platform. The response rate of the new platform will be compared with the conventional BPSU platform. Feedback from service users and notifying clinicians will be collected.

### 4.5 Study impact

#### 4.5.1 Clinical practice

This study will raise awareness of neonatal stroke among healthcare professionals looking after babies. This is important as most healthcare professionals will see only a few cases of neonatal stroke in their career, leading to potential delay in recognising the often-subtle initial signs of neonatal stroke. This study will also explore the variation in practice across UK and Ireland to standardise the investigation and management of neonatal stroke.

#### 4.5.2 Patients and families

This study will provide families caring for babies with neonatal stroke with up-to-date information about the condition including outcome at 2 years of age. This will consolidate information given by healthcare professionals and alleviate some anxiety families may have when first being informed of the diagnosis and caring for babies with neonatal stroke.

#### 4.5.3 Public health

This study will estimate the burden of neonatal stroke. Healthcare professionals and policy developers will be able to use this information to better allocate resources in supporting babies with neonatal stroke.

#### 4.5.4 Scientific community

Neonatal stroke is a distinct entity from childhood or adult stroke. It is multifactorial due to an interplay between the maternal and foetal circulation as well as postnatal factors. There is much to learn on both the mechanism of injury and recovery [31]. Earlier detection may improve outcomes. This study will provide further insight into the epidemiology of neonatal stroke and help inform future studies looking at prevention and treatment options.

From a surveillance study methodological point of view, the study will evaluate the feasibility and success of using a dedicated online platform for the collection of data on rare paediatric conditions.

## Supplementary Material

Supplementary Figure
