# Supplementary material for: Neonatal stroke surveillance study protocol in the United Kingdom and Republic of Ireland
Source: Open Med (Wars). 2022 Sep 5;17(1):1417–24. doi: 10.1515/med-2022-0554 (PMC9449691; doi:10.1515/med-2022-0554)
Supplement: Supplementary Figure [file med-2022-0554-sm.pdf]

Supplementary material

S1 Supplemental material 1: Initial questionnaire

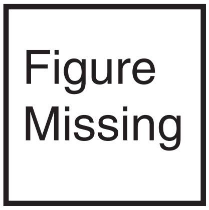

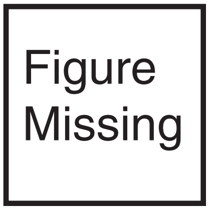

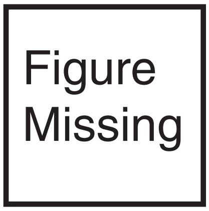

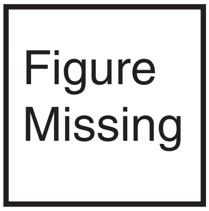

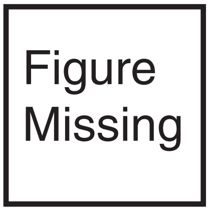

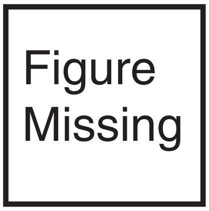

S2 Supplemental material 2: Two-year follow-up questionnaire

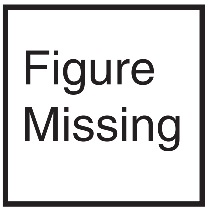

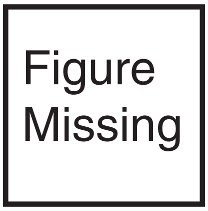

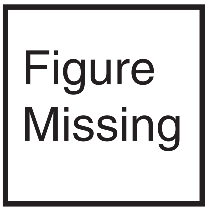

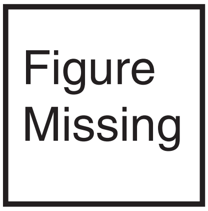

S3 Supplemental material 3: Initial questionnaire (Northern Ireland)

Figure Missing

Figure Missing

Figure Missing

Figure Missing

Figure Missing

Figure Missing

S4 Supplemental material 4: Two-year follow-up questionnaire (Northern Ireland)

Figure Missing

Figure Missing

Figure Missing

Figure Missing
